# Vietnamese mother's conceptions of childhood overweight: findings from a qualitative study

**DOI:** 10.3402/gha.v9.30215

**Published:** 2016-03-29

**Authors:** Loan Minh Do, Viveca Larsson, Toan Khanh Tran, Huong Thanh Nguyen, Bo Eriksson, Henry Ascher

**Affiliations:** 1 Outpatient Department, National Hospital of Paediatrics, Dong Da District, Hanoi, Vietnam; 2 Section for Epidemiology and Social Medicine (EPSO), Department of Public Health and Community Medicine, Institute of Medicine at the Sahlgrenska Academy, University of Gothenburg, Gothenburg, Sweden; 3 Department of Biomedicine and Public Health, School of Health and Education, University of Skövde, Skövde, Sweden; 4 Family Medicine Department, Hanoi Medical University, Hanoi, Vietnam; 5 Faculty of Social Sciences, Behaviour and Health Education, Hanoi School of Public Health, Hanoi, Vietnam; 6 Health Metrics, Department of Public Health and Community Medicine, Institute of Medicine at the Sahlgrenska Academy, University of Gothenburg, Gothenburg, Sweden; 7 Angered Hospital, Gothenburg, Sweden

**Keywords:** childhood overweight, mothers' conceptions, preschool children, qualitative study, Vietnam

## Abstract

**Background:**

Childhood overweight and obesity is a new and emerging problem in Vietnam. The so far observed prevalence increases have pointed to the need for public health intervention strategies with parents as crucial resources for change.

**Objective:**

The aim of this study was to understand mothers’ conceptions of childhood overweight.

**Design:**

Four focus group discussions were conducted with a total of 33 mothers of preschool children, 4–6 years old, living in urban and rural districts of Hanoi, Vietnam. The discussions were audio taped and transcribed verbatim. The obtained data were analyzed using the principles of phenomenography.

**Results:**

Four main categories with 13 subcategories emerged in the process of analysis. The first category, called ‘Concept of overweight’, contained mothers’ views on childhood overweight. A major concern was the negative aspects of overweight such as impaired social interaction and health problems. The second category, ‘Identification of overweight’, described the ways mothers use to recognize overweight in children: own experience, growth chart, and public or health care system's information. The third category, ‘Causes of overweight’, showed mothers’ understanding of factors possibly contributing to overweight development: unhealthy food and lifestyle, genetic susceptibility, parent's lack of knowledge, and limited time to take care of children as well as economic improvement. The fourth category, ‘Management of overweight’, described the ways mothers use to manage a child's weight problem: control of their food intake, increasing their physical activity, and encouraging their child self-control. However, they find such strategies difficult to implement and their intentions are sometimes challenged by the child's grandparents.

**Conclusions:**

The study gives an understanding of the mothers’ conceptions of four important and practically useful aspects of overweight in children. The findings highlight the roles of media and the health care system in enhancing a social awareness of the problem and the need for prevention. Growth charts need to be used more regularly and consciously in child health care for early detection of children at risk and as a tool for information to parents. When designing intervention programs, the entire extended families, especially grandparents and their roles, need to be considered.

## Introduction

The need to control childhood overweight and obesity has lately received increased attention as overweight in childhood and adolescence often persists in adulthood (1). About one-third of overweight children and half of overweight adolescents are predicted to become obese as adults (2, 3). In addition, childhood obesity is associated with several risk factors for later heart disease and other chronic diseases (4, 5) as well as psychological problems (6, 7).

At the individual level, childhood eating habits and lifestyle could be the most important immediate factors contributing to overweight development (8). Diets with high fat and sugar content, and inactivity increase the risk of becoming overweight (9, 10). Parent's understanding of the health risks of childhood overweight in combination with recognition of their child's weight status can motivate them to promote a healthy lifestyle and weight for their child. For example, mothers have reported more use of food control and monitoring when they perceived their children as overweight (11, 12). Recent studies have indicated that parents often fail to correctly classify their children as overweight (13–15). In addition, children of parents who do not consider childhood obesity as an important problem have a significantly higher risk for childhood obesity than other children (16).

Vietnam is experiencing a nutrition transition and is facing a double burden of malnutrition. During the last decade, although the prevalence of underweight has been reduced remarkably, it still remains as high as 17.5% among children under 5 years in some, particularly rural, areas (17). Meanwhile, the prevalence of overweight and obesity in children is on the rise, especially in the big cities with high socio-economic status (17). Results from the Vietnamese nutrition surveillance in 2014 indicate that the prevalences of overweight and obesity in children under 5 years of age in Hanoi and Ho Chi Minh City, were 4.6 and 12.9%, respectively (18, 19). Childhood overweight and obesity have become public health concerns in Vietnam which underlines the need for development and implementation of public health intervention strategies (20).

Prevention and intervention programs of childhood overweight with the parents as crucial persons and resources for change are promising models (21, 22). Education programs aimed to change parents’ conceptions and practices through improved knowledge of childhood overweight are commonly suggested, based on evidence from previous studies (23–25). Information about parents’ conceptions, especially mothers who are primarily responsible for the care of the child, is however, scarce in Vietnam (26). The aim of this qualitative study was to explore how mothers of preschool children conceive childhood overweight. The understanding obtained from the present study can be used by health professionals and others to help mothers develop ways of recognizing childhood overweight, and enable them to act effectively in managing the problem.

## Methods

### Study design

A qualitative study using phenomenography was performed. Phenomenography was developed in the early 1970s for the analysis of studies on human learning (27). The basic assumption is that a phenomenon of interest has different meanings for different persons and the analysis aims to describe this variation qualitatively (28). In phenomenography, conceptions refer to the qualitatively different ways in which a certain phenomenon and some aspects of reality are understood as meaningful to the study participants (29). This is the definition of ‘conceptions’ used in the present text. ‘Categories of description’ are used to characterize these conceptions. Focus group discussions (FGDs) were used as a way to collect information from participants.

### Procedure and participants

Participants were mothers of preschool children (age range from 4 to 6 years old) living in two Health Demographic and Surveillance Sites (HDSS), urban DodaLab (Dong Da district) and the rural FilaBavi (Ba Vi district), Hanoi, Vietnam (30). The mothers were selected purposively and invited to participate in the FGDs. The main purpose of the strategic selection process was to have large diversities with respect to mothers’ and children's weight status, mother's age, education, and work as well as family economic status in order to obtain broad ranges of views on childhood overweight. The databases from the HDSS collected in 2012 were used to identify 78 suitable participants. Four field workers who had been involved in household surveys in HDSS were trained about the purpose of conducting the FGDs as well as about the need for informed consent and the rights of participants. These field workers contacted and met the mothers from the list of suitable participants and informed them about the study. In total, 33 mothers, 18 urban, and 15 rural mothers (21–42 years old), gave their consent to participate.

### Data collection

Four FGDs, two in urban and two in rural areas, were conducted at community health centers in January–February 2015. The groups included 7–10 participants, who all at that time had at least one daughter or one son aged 4–6 years. The FGDs were composed of mothers with different backgrounds and livelihoods. Two researchers, authors of the paper (LMD and TKT), facilitated the group discussions, one was the moderator, and the other was the assistant taking notes. The discussions lasted about 60–90 min and started with an introduction of the topic and the purpose of the study. The importance of active participation, contributions from all participants and the freedom to make any statement were emphasized. The word ‘childhood overweight’ was introduced as a state where a child, appears to weigh more than is, subjectively seen, normal. No technical definition was presented and no distinction between overweight and obesity was made. The discussion was then started asking the group members to discuss this phenomenon without mentioning any particular topics. Some prompting questions were used in focus groups when necessary to capture ideas of the participants, for example, ‘How could you describe your child's weight?’, ‘What do you think about overweight children?’, and ‘Why do you think some children are overweight but others not?’

A pilot study, later not included in the analysis, was performed with 11 mothers of preschool children in urban and rural areas to ensure that the language used for the introduction and moderation of the discussion was understandable.

The discussions were audio taped. The recordings were transcribed verbatim in Vietnamese and checked by two of the researchers (LMD, TKT) to make sure that the audio matched the transcript. Only the quotations shown as illustrations in this paper were translated to English.

Anthropometry of the children and the mothers was extracted from the study conducted in HDSS in 2013 (8). For children, overweight (including obesity) was defined using WHO standards (31, 32). For mothers, overweight (including obesity) was defined as BMI ≥25 kg/m^2^.

### Data analysis

A phenomenographic approach was used to identify categories and subcategories in the transcripts (see Table 1) (33, 34) following the seven steps as described by Dahlgren and Fallsberg (35). First, all transcripts were carefully read by the researchers (LMD, VL, TKT) to become familiar with the material. Second, all statements considered meaningful from the discussions were also selected by the researchers independently and abbreviated into excerpts, as far as could be done without loss of information. Third, similarities and differences between the selected excerpts were identified. Fourth, similar statements were grouped into internally homogenous but externally different categories. Fifth, the essence of the similarity within each category of answers was described. Sixth, the categories were adequately named to highlight these essences. The seventh step was to make a comparison of the obtained categories with regard to similar and different characteristics.

**Table 1:** Overview of mothers’ conceptions of childhood overweight

Category	Subcategory
Concept of childhood overweight	Chubbiness is good
	Overweight is a minor problem
	Impairment of social interaction and health problems
Identification of childhood overweight	Mothers’ own experience
	Using growth chart
	Using information from media or health care system
Causes of childhood overweight	Unhealthy lifestyle
	Heritability
	Negative impact of economic improvement and livelihood
	Food containing growth stimulants
Management of childhood overweight	Control of food intake and increase in physical activity
	Encouraging child's self-control
	Challenge by grandparents

Suitable supporting quotations to be presented verbatim were identified. The categories, subcategories, and supporting quotations were discussed between all researchers until agreement was reached and final decisions were made.

### Trustworthiness

In the present study as well as other qualitative research, the creation of trustworthiness is important. The emerged categories must stem from the discussions and analysis of data without being influenced by the views and preconceptions of the researchers. To ensure this as far as possible, one researcher identified the categories from the material while a co-examiner started from the obtained descriptive categories and re-read, comparing the quotations of the discussion, within and between the categories. The quotations in the study were also checked by the researchers independently of each other to ensure that they all accurately derived information from the original material. The researchers who conducted the analysis had been present in the discussions and could refer to nuances and meanings in the discussion.

### Ethical considerations

The mothers participated voluntarily, had the possibility to withdraw at any time, and were able to get advice on childhood obesity from medical doctors in National hospital of Pediatrics if they wanted. Ethical permission (140/HMU IRB) was received from the Scientific and Ethical Committee of Hanoi Medical University on 24th December, 2013.

## Results

Among the 33 mothers participating in the FGDs, 12 were overweight. The levels of the mothers’ education were secondary school (nine mothers), high school (12 mothers), and above high school (12 mothers). Sixteen mothers had overweight or obese children.

Mothers’ conceptions of childhood overweight were described by 13 subcategories and 4 categories, which emerged in the analysis of the information obtained in the discussions (Table 1).

The following results are presented first with a general description of category, followed by corresponding subcategories and supported quotations from material.

### Concept of childhood overweight

This category describes the views of the mothers about childhood overweight. What do they think of and believe about different aspects of overweight in children? In the FGDs, it became clear that conflicting connotations of overweight existed.

#### Chubbiness is good

Some mothers shared the thought that they wanted their children chubby and liked this appearance. Chubby children look cute and lovely and the mothers could get compliments from others. Mothers also considered it possible that chubby children can be healthier than other children. When caring for sick children, they observed that chubby children who are ill recovered faster than underweight children. Having such a child makes a mother feel happy and secure.

Vietnamese mothers, from a psychological aspect, always want their children to look a little bit chubby. Only when the kids get compliments for their ‘cute’ chubbiness do the mothers feel assured. (FGD_4)

#### Overweight is a minor problem

A few mothers stated that since their overweight children were doing well like their peers, they thought that overweight was a minor problem. They considered overweight children as normal except that they are bigger than their peers. This was however, considered as nothing to be worried about.

He's fatter than his peers, but he still runs and plays with his friends, has good appetite, gets good grades, has good handwriting, just like any other child. The only difference is that he is bigger. (FGD_ 2)

#### Impairment of social interaction and health problems

Most mothers viewed overweight in negative or even shameful ways. Obese children were considered in contrast to prevailing ideals of being good looking and involved in activities.

It feels bad to be fat. Slow actions, ugly appearance. Being fat in a crowd is embarrassing. (FGD_1)

Overweight children were also considered to be limited in being able to participate in some social activities, such as playing with peers and being in art performance teams, due to health problems and body image. Teasing or bullying because of the appearance of a child is considered to be a possible obstacle especially in urban areas.

It's more difficult for him (obese child) to be involved in physical activities with his friends. He gets tired very quickly and sweats a lot, which makes him even more reluctant to be active. (FGD_2)

Health effects related to overweight were the dominant concerns for the mothers and was lively discussed. Some types of problems, such as high blood pressure, cardiovascular disease, diabetes, high cholesterol levels, and fatty liver disease, were the ones most often mentioned.

I think diabetes, many cardiovascular diseases, blood fat, liver fat … all of them has to do with overweight. (FGD_2)

Moreover, mothers emphasized that the treatment for diseases such as diabetes was more difficult in children than in adults. Children are not aware of the disease and that makes it difficult for them to strictly adhere to the treatment.

Diabetes in children is more difficult to treat than adults because it's very hard for children to be on a diet. (FGD_4)

### Identification of childhood overweight

The category describes how a child could be identified as overweight by the mother herself. The main approaches to assess the weight status were the mother's own judgments of the outward appearance of the child, comparison with other children, using official standards (growth chart) and public information in media or from the health care system.

#### Mothers’ own experience

Mothers defined abnormality in weight through comparison with other children in the same age. They make visual estimations from the child's body appearance such as the shape of face, belly, arms, and legs. Mothers believed that if these parts of a child looked bigger than the parts of their peers, or older children, the child must be overweight. In addition, some mothers would become concerned about a child's weight when they have to buy clothes for their child with a much bigger size than should be expected. Some mothers also judged the child's movements and activity level and came to the conclusion that if they saw children with a big size and slow movement, these children were considered overweight.

A child has a big bottom, round and fat face, moves slowly. In general, he is not as dynamic as other kids. That's how I tell the kid is obese. (FGD_2)

#### Using growth chart

Instead of judgment by appearance, mothers, mainly urban, use growth charts which are available at community health centers or the Internet, to assess the weight status of their child. They compare their child's weight and height with the reference curves. Mothers expressed their beliefs in this method for classification of child's weight status. They also commented that the growth chart was easy to use.

In the chart, there are lines going up and down, yellow lines, green lines. Yellow lines mean normal or risk group 1 or 2 or obese or malnourished. The charts are clear. (FGD_4)

#### Using information from media or health care system

Information provided by mass media, such as TV, newspapers, online magazines, and the Internet, helps some mothers to become aware of child overweight. Some useful shows on public service TV, for example, ‘A healthy mom–healthy child’, ‘A healthy life’, or the O^2^ TV channel which specializes in health issues were mentioned. The mothers reported that they learn and practice the instructions and advices on nutrition from such resources.

Watching TV, we can see that obesity is very common now, not only in Vietnam but also in the world. For Vietnamese standard, a 2–3 year-old weighing 13–14 kg is certainly overweight. Second graders (7 years old) normally weigh 20–21 kg but if it's 30 kg, it's definitely obesity. (FGD_2)

Some mothers recognized that the child was overweight or obese after receiving information from kindergarten, school or the community health center where weight status of children was checked.

The school doctor or nurses at the community health center warned me that he was overweight. (FGD_3)

Almost all mothers had a strong confidence in health professionals in matters regarding their child's weight. Although mothers could seek advices from different sources, only information from medical doctors is believed to be trustworthy enough to judge if a child is overweight or obese and to explain how this can affect the child's development.

Of course I believe in doctors; that's why I bring my child to the hospital. You can Google symptoms on the Internet but you can't trust this information completely. You still need to visit doctors. (FGD_4)

Only doctors know how a child's normal development should be. We can't know such a thing. What doctors say during check-up is the most accurate. (FGD_1)

### Causes of childhood overweight

The reasons for overweight were understood by the mothers to be the result of several factors such as eating habits, sedentary behavior, and genetic predisposition as well as economic and environmental factors. For individual children different combinations of such factors were perceived to be responsible for the development of overweight and obesity.

#### Unhealthy lifestyle

The mothers in our study agreed strongly that unhealthy lifestyle, including eating habits and inactivity, was the main cause leading to overweight in children. They observe that obese children eat too much, especially rice, fatty meat, sweets, confectionery and snacks. Several mothers said that overweight children were also considered to have a good absorption and digestion of food.

I (mother) notice that my child is quite obese. His appetite seems boundless. He likes to eat everything. (FGD_1)

It's probably because of their good absorption. Like my child, she eats quite moderately but is still bigger than her peers. (FGD_4)

The overweight children were also described as lazy and inactive which some mothers related to the city lifestyle. Some mothers also mentioned the inactivity due to playing with phones and iPads, contrasting that to activity when children play running around in the yard.

Children in big cities always sit in one place, playing with phones or iPads. They don't run around and play. (FGD_4)

Mothers realized that children involved in regular physical activities such as running, playing, jumping etc. had healthy weight. A rural mother said that:

All my children are very active. They run around all day. As a result, they are all healthy. None of them is overweight. (FGD_2)

#### Heritability

Another aspect mentioned was that often several overweight or obese people, for example, mother, father, and relatives, were found in the same family. The mothers believed that genetics could be an explanation for some but not all of this. It might also be due to sharing the same food environment or parenting style.

Genes are also responsible. Like my family, everyone is big. We are sometimes jokingly called ‘the bear family’. (FGD_4)

Genes have an impact but it's not that significant. Why is everyone in a family obese? It's also because of the diet they share, not just because of the family gene. (FGD_4)

#### Negative impact of economic improvement and livelihood

The mothers perceived that improved family economy influenced the child's weight. Better economy means that parents could buy more food for the children, offer favorite foods to them and let them eat whatever they want. These might all be risks of becoming overweight.

When families are wealthier, children can eat pretty much whatever they want, which may cause obesity. (FGD_3)

Some mothers acknowledged mistakes of their own that contribute to the child's overweight. Parents’ lack of time gave the children an opportunity to eat freely. Lack of knowledge could be a reason not to feed the children properly. Mothers blamed the busy life nowadays and, especially urban mothers that they had to work hard which takes away a lot of their time.

… Because I am too busy in our daily life. For example, I just give our child meat or fish without any vegetable because it's quicker that way. I have other chores to take care of. (FGD_2)

#### Food containing growth stimulants

The mothers suggested that food containing growth stimulants given to, for example, chicken, pork, beef, and vegetables might create overweight. Most mothers thought that such stimulants, used to speed up growth of animals and vegetable for commercial purpose, could influence humans eating them in the same way. It was mentioned that it is quite difficult to distinguish between food containing stimulants and food without stimulants.

In urban areas, meats and vegetables in the daily diet usually have unsafe hormones (‘thuoc tang trong’). In the countryside, since families grow their own vegetables, raise their own livestock, the food is safer, cleaner. I think that's why obesity is less common in rural areas. (FGD_1)

### Management of childhood overweight

The mothers’ conceptions of how overweight children could and should be managed in everyday life were mainly described in terms of how children and parents together can handle overweight problems. Control of food intake and increase in physical activity were the main strategies that most mothers wanted to use. However, they found such strategies difficult to implement and that they were sometimes challenged by grandparents.

#### Control of food intake and increase in physical activity

The mothers in the study were aware that food intake needs to be controlled with respect both to quantity and quality. They try to lower the amount of rice in each main meal. Mothers especially try to avoid sweet and fatty food as well as fried food which they consider to provide too much energy. They emphasized that the reduction of food intake should be made bit by bit to make it easier. Some mothers discussed a combination of food intake restriction and increase in physical activities such as walking, running, swimming, playing badminton, and so on, which they considered as a good method. Mothers even understood how these methods work.

The only way is to control his diet and increase the physical activity. Changing his diet from milk with sugar to milk without sugar, 3 bowls of rice to 2 bowls, 2 pieces of fried chicken to 1 piece. I don't want to make any radical change because he will crave food and eat even more if he gets the chance. (FGD_3)

First, physical activity uses up energy. Second, reduced food intake means reduced energy intake. When energy intake doesn't exceed energy usage, the child will maintain weight or lose weight. (FGD_4)

However, most mothers found it difficult to control children's eating, especially for children who were always hungry. The mothers described that if they reduce the amount of food intake at the moment, the child later ate more than before or found a way to eat when the mother was away. In addition, some mothers felt sorry for the children when they had to be on a diet, which made mothers having a difficulty in denying the children food.

I can make him stop eating now but after playing with his friends for a while, he eats even double or triple more. (FGD_3)

It is quite miserable for my child to be on a diet because of obesity. I, as his mum feel bad to him if I say no when he is still hungry. But if I let him eat he will become obese, even suffering from other things, disease and personalities. (FGD_4)

Mothers suggested that avoiding placing food, especially junk food, in front of children might enhance the effectiveness of the attempts to decrease food intake as it helps children to resist the temptations.

I hide all the unhealthy food from my children and only allow them to drink boiled water or juices. (FGD_4)

Although choosing food restriction to manage children's weight, mothers still questioned its effectiveness and worried about unfavorable influence on the child's health. They expressed that:

In fact, it (restriction of food intake) does not work, my child still puts on weight. (FGD_1)

Dieting methods can help children lose weight but their health might be negatively affected. (FGD_4)

#### Encouraging child self-control

Encouraging children to self-control was a way mothers found effective although it is considered better mainly for older children. When understanding the weight and weight-related problems, children could act appropriately without parents’ promotion. Mothers mentioned that experience from being teased at school could motivate them to control the weight.

I recognized that my child was a bit overweight when she was in the third grade, so I started telling her to control her diet or else she would get fat. She understood and ate less without me forcing her. (FGD_3)

Mothers suggested that changes should be made by the child itself, but with the assistance of the family. They encourage children to do some activities that their children are interested in, for example, playing sport, joining a dancing club, painting, and so on. Mothers believed that once children are enthusiastic about something, they could concentrate on it without thinking about food.

Going to art class, chess class - focusing on doing something will distract them from thinking about food. (FGD_3)

#### Challenging by grandparents

One-third of the participants, mainly urban mothers, mentioned that managing child overweight could sometimes be challenged by grandparents. Control over child eating habits in some cases became dependent on the grandparents’ involvement as they had their own opinions about what is not good and unnecessary for the grandchild. However, mothers often realized that not all grandparents’ views correspond with the thinking of today's society and its view of overweight and obesity. Grandparents could be happy for having a heavy grandchild, in conflict with the mothers’ view.

I live with my parents. When I recognized that my kid was overweight and told him to stop eating so much, my mom yelled at me: ‘He's not fat. Just let him eat whatever he likes. (FGD_3)

## Discussion

To our knowledge, this is the first qualitative study in Vietnam exploring mothers’ conceptions of childhood overweight. The main findings are that there are really quite different conceptions of childhood overweight among mothers. The participants reported that both subjective methods, like simply looking at a child, and objective, using quantitative templates, were used to identify overweight in children. Mothers thought that childhood overweight was the result of a complex set of factors. Different measures for prevention and management were suggested. The need for involvement of entire families, particularly the grandparents as well as children themselves, in managing childhood overweight was emphasized.

Although the conceptions of childhood overweight varied among mothers in the study, two main opposing views were found. On the one hand, the mothers believed that a chubby child was a lovely and healthy child. This notion has persisted in the Vietnamese culture for years (36, 37). Vietnam in the past underwent long periods of war and famine. People, especially children, suffered from a lack of food resulting in high prevalences of underweight in children over long periods of time. These unpleasant experiences might, in the contemporary society, make parents feel more secure when they have a chubby child. Similar opinions like ‘a bigger infant is a better infant’ have been seen in other countries with different cultures, history, and socioeconomic status, such as the United Kingdom and the United States (38, 39).

Diet, exercise, weight, and beauty can be important components of common health discourses today (40). In a study by Craig in Vietnam, the informants discussed how being large or slightly fat could be regarded as embodiment of strength and representing status, however with different views in urban and rural contexts as well as in old and young people (40). This thinking may, to some extent, explain the differences between the views of today's mothers about overweight children and those of older relatives who grew up under different economic conditions.

According to the mothers in this study, it is good if the child is chubby, however, without making any clear distinction between chubbiness and overweight. A chubby child could well be an overweight child. This suggests that the delimitation between ‘chubbiness’ and ‘overweight’ needs to be clarified.

On the contrary, mothers were concerned about excess weight and its consequences, including psychological, social, and especially health problems. Our results show that experiences of being obese or observing persons with obesity make mothers aware of such effects. In other words, there is persuasive evidence even in daily life that can make mothers understand the negative aspects of overweight. The Vietnamese government has recently identified overweight as a problem, and has presented it into public concern as ‘the double burden of nutrition’, undernutrition and overnutrition (41). The government thus has taken action to control overweight and obesity by ratifying a National Nutrition Strategy for 2011–2020 (20, 42). According to this, mass media is thought to play a vital role in raising awareness and improving knowledge about overweight and obesity in the general population. For example, the Vietnamese nationwide O^2^ TV-channel broadcasts shows containing health information, which contributes to mothers’ perceptions of childhood overweight. In addition, mothers’ beliefs and attitudes about overweight are influenced by social beauty ideals that change over time. Thinness has recently become a symbol of beauty in Vietnam, leading to a fear of being fat.

Quantitative studies have indicated that a high percentage of parents fail to correctly classify children's weight, especially their overweight status (15, 25, 43, 44), but the reasons for misperception have not been explained. The present qualitative study gained some insight into this. Most mothers in our study based their judgment of the child's weight status on his or her appearance or on comparisons with other children. This will often be an incorrect method except for extreme cases. Similar observations have been made in some high-income countries such as Australia, Germany, UK, and USA (23, 24, 45, 46). These methods are subjective and should be complemented by more precise tools, such as growth charts. Some mothers left the assessment of their child's weight status to school or to the health care system. This could reflect that they are not confident with how they determine their child's weight status themselves but may also refer to a strong tradition of relying on professionals and professionalism. The results of the present study suggest that mothers need help to identify children with overweight or children at risk for developing overweight. Considering that mothers highly appreciate professional knowledge and judgments, discussions with mothers about their child's growth is important at all levels of the health care system.

As opposed to a subjective evaluation of comparison with peers, some mothers, especially urban mothers, trusted growth charts, as an objective assessment of child weight status. The growth chart is a useful reference for the growth of children and can be used to screen for overweight and obesity problems. In contrast to the study by Jain et al. (23) in which mothers shared their dislike and distrust of the growth chart, almost all mothers who have used growth chart in our study, urban as well as rural, found it to be a meaningful method to detect potential weight problems. This may again refer to trust in professionalism and written information (47). The WHO international growth standards (31, 32) are easily accessed in health centers, but downloading from the Internet is also a frequent practice in Vietnam. The growth charts show the child's normal development and its variations in a simple way that everyone can understand and use. The trust in growth charts by mothers suggests that their use should be encouraged in the health system and the community for screening the growth of children.

Aside from growth charts, mothers put their trust in health providers in solving their child's health problems. This indicates that the role of the health care system in general and health professionals in particular is important for managing the obesity ‘epidemic’. In Vietnam, access to all levels of the health care system is easy and convenient without need to make any appointment, which allows parents to get advice whenever they feel there is a need. Therefore, professional monitoring of the weight of the child is quite possible if parents or caregivers are aware of the importance of following their children's weight. Health professionals could discuss and advise mothers to what they could do to improve the situation with their overweight children.

Similar to previous qualitative results on the perceptions of causes of overweight, mothers in the present study associated excess weight to overeating, less physical activity, and genetics (23, 45, 46, 48). However, in contrast with results from studies in other countries, the Vietnamese mothers also indicated the possible influence of socioeconomic factors on the development of childhood overweight. The recent rapid social and economic changes in the Vietnamese society could be an underlying reason. Vietnam's economic improvement during the past 10 years (49) has led to better living conditions, especially in big cities. On the contrary, the improving economy can also result in negative impacts on lifestyle, for example, unhealthy habits, sedentary behavior, and growing economic, social, and health gaps in the community (50). According to the mothers, the improved economy allows families to spend more money on food and this opens wider choices. Children can eat pretty much whatever they want, including unhealthy food. Together with a lack of knowledge of the child's feeding practices and limited time for taking care of children, this creates new risks to develop overweight. Relations between parents’ time pressure, financial stress, and healthy lifestyles of children have been observed in the Nordic countries (51). It is important that caregivers and children get appropriate knowledge on healthy foods to avoid risks for food and overweight-related diseases.

Food possibly containing growth stimulants as a cause of overweight was another issue identified during the discussions. The use of growth stimulants in animal husbandry and farming is quite popular in Vietnam today and profitable for farmers. Mothers, who are farmers, admitted that although they use these stimulants, they believe that they have not eaten food and vegetable containing it. To our knowledge, there has not been any official scientific conclusion about the effects of such food on the health in general and in particular on overweight development. This points to the need for scientists as well as relevant authorities to investigate this issue.

To manage children's weight, based on the understanding of the causes of overweight, the mothers suggested reduction of food consumption, in ways similar to that of mothers from other countries (23, 48). However, the mothers also indicated that the effectiveness of food restriction is not likely to be high, which is in line with other qualitative studies (23). The lower effectiveness was said to be due to the seemingly unlimited appetite of overweight children, whereas the mothers found it hard to refuse children food and pitied children who were on a diet. In addition, the mothers were anxious about the impact of diet on the child's health. Nutrition is essential for growth and development of children, thus mistakes on children's diet may result in health consequences later. Mothers or caregivers need to be given advice carefully by dieticians or other skilled health professionals about child diet which should be designed appropriately for child age and for any specific child and situation.

Although mothers were aware of the relation between sedentary lifestyle and overweight, and tried to encourage physical activity among children to control their weight, the mothers often did not manage to modify sedentary behavior. There are many recommendations saying that television and ‘screen time’ should be limited to less than 2 h per day (52). Furthermore, there is evidence that targeting activity and inactivity may be effective in the treatment of childhood obesity, especially television viewing (53).

Our data reveal that not only parents but also children and grandparents need to be involved in management and prevention of overweight. Involving the children themselves is important but adult support from the surroundings (family, school) is essential. In the present study, mothers said they believe that once children conceived their weight problem, they will themselves find a motivation to control their diet and weight, especially older children. Grandparents did not perceive their grandchild as overweight, and therefore to a certain extent support overweight in children or challenge mothers about their decision for child eating and possible restrictions. Similar observations have been made in previous studies conducted in America (23, 48). This suggests that obesity prevention messages should be aimed not only to parents but also the extended family members particularly grandparents.

### Methodological comments

Phenomenography is a qualitative approach well suited to provide qualitative descriptions of the variation in mothers’ ways of understanding childhood overweight. As it is important that the descriptions do well reflect such variations, participants in the study were strategically selected and groups were composed to contain mothers with differing characteristics. The larger the number of persons providing information is, the more of variation is likely to be observed but there is really no way to formulate strict criteria for any ‘necessary’ number of informants. The concept of saturation is often used in qualitative research and means that the researchers consider that a stage is reached where additional information will add only marginally to the knowledge. In the present study, the number of participants is moderate and statement of saturation cannot be made with certainty. Rather than claiming that the study missed very little of the range of conceptions, we argue that the findings actually made are definitely of interest and add substantially to the knowledge in the field especially in the Vietnamese context.

One of the main questions in qualitative research of persons is the choice between making in-depth interviews or conducting FGDs. Some informants would certainly provide better information in interviews than in the group where there is a risk that some come to dominate and others remain silent. Such tendencies were actively counteracted in the present study.

Our interest has been the variation in the way mothers conceive overweight in children, not to do systematic comparisons between groups of mothers. Future studies, using mixed-methods to explore differences between urban and rural, influence of mothers’ education, etc., should be considered.

## Conclusions

The presented study describes mothers’ conceptions of childhood overweight. The most common concerns mentioned by the mothers were the concepts of childhood overweight, identification of overweight, causes of overweight, and management of overweight. These appeared to be the most essential aspects of childhood overweight. Although the results of a qualitative study cannot be used to make formal generalizations, we consider the findings trustworthy enough to support some suggestions for prevention. A systematic use of growth charts needs to be encouraged for screening of risk for unhealthy growth. To effectively manage overweight, the whole extended families, least of all grandparents, should be involved. Mass media and the health care system together need to be involved for increasing the awareness of overweight in the society.
